# Supplementary material for: Biomechanics of running: A special reference to the comparisons of wearing boots and running shoes
Source: PLoS One. 2022 Jun 24;17(6):e0270496. doi: 10.1371/journal.pone.0270496 (PMC9231798; doi:10.1371/journal.pone.0270496)
Supplement: S1 Table — (DOCX) [file pone.0270496.s002.docx]

| Marker | Definition | Type | Static | Dynamic |
| --- | --- | --- | --- | --- |
| C7 | C7 spinous process | Anatomical | * | * |
| T10 | T10 spinous process | Anatomical | * | * |
| CLAV | Right sternoclavicular prominent | Anatomical | * | * |
| RACR  LACR | Right/left acromion process | Anatomical | * | * |
| RLEL  LLEL | Right/left lateral elbow (humeral lateral epicondyle) | Anatomical | * | * |
| RFAradius  LFAradius | Right/left lateral wrist (radial styloid process) | Anatomical | * | * |
| RASI  LASI | Right/left anterior superior iliac spine | Anatomical | * | * |
| RPSI  LPSI | Right/left posterior superior iliac spine | Anatomical | * | * |
| RHJC  LHJC | Right/left hip joint center | Virtual | * |  |
| RTH1-RTH4  LTH1-LTH4 | a 4-cluster on right/left thigh | Tracking |  | * |
| RLFC  LLFC | Right/left lateral epicondyle of femur | Anatomical | * | * |
| RMFC  LMFC | Right/left medial epicondyle of femur | Anatomical | * |  |
| RKJC  LKJC | Right/left knee joint center | Virtual | * |  |
| RTB1-RTB4  LTB1-LTB4 | A 4-cluster on right/left tibia | Tracking |  | * |
| RLMAL  LLMAL | Right/left lateral malleolus | Anatomical | * |  |
| RMMAL  LMMAL | Right/left medial malleolus | Anatomical | * |  |
| RAJC  LAJC | Right/left ankle joint center | Virtual | * |  |
| RCAL  LCAL | Right/left calcaneus tuberosity | Anatomical | * | * |
| RMT1  LMT1 | Right/left 1^st^ metatarsal head | Anatomical | * | * |
| RMT5  LMT5 | Right/left 5^th^ metatarsal head | Anatomical | * | * |
| RFF  LFF | Right/left forefoot (mid MT1 and MT5) | Virtual | * |  |
