# Supplementary material for: Biomechanics of running: A special reference to the comparisons of wearing boots and running shoes
Source: PLoS One. 2022 Jun 24;17(6):e0270496. doi: 10.1371/journal.pone.0270496 (PMC9231798; doi:10.1371/journal.pone.0270496)
Supplement: Attachment — Submitted filename: Response to Reviewers.docx [file pone.0270496.s007.docx]

Dear editor and reviewers,

Thank you for the kind comments. We tried our best to take all comments into account. Additionally, several minor typos were found during revision and fixed accordingly.

**Editor comments:**

1. Please ensure that your manuscript meets PLOS ONE's style requirements, including those for file naming.

The file naming was corrected as requested.

2. We note that you have indicated that data from this study are available upon request. PLOS only allows data to be available upon request if there are legal or ethical restrictions on sharing data publicly.

The dataset was uploaded (“DataSet.zip” (S2_File)) as requested. The file contains OpenSim model, setup, and output files.

**Reviewer #1:**

1) L22-23: "Reduced ankle range of motion was the only kinematic effect of running with boots"? This is not clear; please rephrase.

Thanks for the comment. The sentence was rephrased as follows: “The only effect of boots on lower limb joints kinematics during running was a reduction in ankle range of motion”.

2) L35-36: "Lower limb injuries during running are very common in the active population with an annual incidence of between 20 to 80% [1]. " the presented percentage of injuries linked to running activity is a bit general and somehow unrealistic in the upper bound (80%). Could you please be more specific and clear on this point, especially how can 80% of runners may get injured? This is a very important point since some readers/researchers may use this paper later as a reference.

The percentages were belong to (<https://doi.org/10.1136/bjsm.2006.033548>) (between 19.4 and 79.3%). This is a fact that the prevalence of running-related injuries might be too high. However, we changed the reference based on a more recent article as follows: “with a 2-year incidence of 66% (73% for women and 62% for men)” (REF: <https://doi.org/10.1177/0363546518773755>).

3) L36: "A total of 28 lower extremity conditions have been reported in the literature as injuries...". Could you please replace conditions with alterations?

If I understand this comment correctly, I replaced the term “conditions” with “alterations”.

4) By the end of the introduction, it is better to emphasize the importance of the scientific question that you are trying to answer via this study. In other words, what is the added value of the proposed study?

We mentioned the importance of our study in the last paragraph of the introduction. However, the paragraph was modified as follows: “Identifying the biomechanical alterations in lower limb joints while running with boots may help to better understand the underlying mechanisms of the related injuries. The results of this study may have clinical implications for treating people with footwear-related injuries who frequently use boots. Furthermore, this may aid to develop the footwear design in order to reduce the risk of both running and footwear-related injuries. Given that there are few studies in this field, therefore, the purpose of this study was to compare the temporal-distance gait variables, ground reaction forces (GRF), kinematics, and kinetics during running with boots compared with running shoes”.

5) It's important to add a picture of the used shoes and boots. This may help the readers to draw some additional conclusions.

A picture of boots and shoes was added as Fig 1.

6) Here is not clear if the authors have used different sizes of the boots/shoe or just one size (42 EU), as indicated in Table 1. An explanation needs to be addressed if it is one size.

Thanks for the comment. The sizes in Table 1. were omitted. We used 3 sizes (from 41 to 43 EU) to fit all participants’ feet during data collection and the values reported in Table 1. belong to the 42 EU size.

7) The authors need to present the adduction-abduction and internet-external knee rotations, which are additional tools for the reader to draw some observations on joint load distributions and muscle activations.

Thanks for the comment. An illustration of tibia motions relative to the femur in frontal and transverse planes was added (Fig2) for this purpose. Please let me know whether this is exactly what you are asking for or not. Since the knee joint was defined as a revoulute joint in the Rajagopal model (the model in OpenSim), we were able to measure the sagittal plane movement (kinematics) only.

8) Here not clear why the authors have ignored muscles activation measurements via EMG or even theoretical optimization via OpenSim; this info may endorse a lot of presented observations in the discussion section.

We couldn’t collect the EMG signals at the time of data collection because the laboratory didn’t have any sound device for this purpose. In terms of OpenSim muscle analysis tool, to the best of the authors' knowledge, the original model (Rajagopal) is robust for muscle analysis tools in OpenSim. However, the model was modified in this study as follows: removing all muscles from the model (in terms of increasing the speed of the workflow) and patella, applying several modifications to the knee joint in order to calculate knee moments in frontal and transverse plans while the parametrized knee was intact (these modifications were made based on Saxby et al. (2016) and Lenton et al. (2018)). Also, the range of motion of all defined coordinates was unclamped (meaning there will be no restriction) which influences the prescribed wrap geometries of muscles. These modifications are an added value for inverse kinematics and inverse dynamics tools, but they may influence the way the muscles force/activation are calculated by muscle analysis tools (CMC or SO). Additionally, the current optimization algorithm in OpenSim assumes tendons are rigid, uses acceleration-matching approach, considers no weight for muscle volume or strength in the optimization cost function, and ignores muscle dynamics and passive elements. These limitations may lead to a poor estimation of muscles activity when compared with EMG signals, refer to the OpenSim forum:

<https://simtk.org/plugins/phpBB/viewtopicPhpbb.php?f=91&t=14556&p=0&start=0&view=&sid=ee032bc98ffe227345ae8a76a535470f>

<https://simtk.org/plugins/phpBB/viewtopic.php?f=91&t=12969&start=0&view=&p=37177#p37142>

Nonetheless, calculating the muscle force/activity using more robust optimization algorithms is more sophisticated which is not possible in the current OpenSim GUI and is beyond our expertise. Also, please note that the generic musculoskeletal modeling is not as robust as subject-specific musculoskeletal modeling (<https://doi.org/10.1371/journal.pone.0262936>). Therefore, this study didn’t take muscle analysis into account.

9) Paper needs to go through minor language editing to avoid some grammatical errors. Herein are some of them were detected:

L199: a comma before which

L207: partly be explained

L216: in boot conditions (The boot condition)

L243: positive ankle

L244: average ensemble

L252: the frontal

L283: a lack

Thanks for the comment, the errors were fixed.

**Reviewer #2:**

Please provide all data in figures.

The data of the figures were provided (Excel files) in the supporting materials (S3-S6).

The way you analyses the data And complete the methods and results section Statistical analysis in not what we expected from this type of study.

Sorry, I’m not sure I understand this comment correctly.

Statistical Parametric Mapping is a statistical tool for continuous data and this tool has been used in many similar studies. For instance (<https://doi.org/10.1080/19424280.2021.18928344>). This tool was used in this study instead of a discrete analysis in order to observe any potential difference in all sub-phases of the running cycle. Also note that the critical t value in SPM is corrected (based on Random Field Theory: <https://doi.org/10.1016/j.jbiomech.2013.07.031>) which is an added value of this tool compared with common Paired t-test statistics.

**Reviewer #3:**

Title: Specify if the shoes are running or everyday shoes.

The shoes were running shoes (based on the manufacturer’s claim). The term “running” was added to the title.

Abstract. Explain what means a greater net contribution (line 30).

It means the overall contribution (activity) of muscles that generate or control a movement (agonist\antagonist muscles). Since we didn’t measure muscle activity directly by EMG, the best way to talk about muscles is by interpreting joint moment and power. Also, this term is common in the biomechanics of movement (e.g. <https://doi.org/10.1016/j.clinbiomech.2009.08.003>) and I’m afraid I couldn’t find an alternative for that.

Introduction. Correct: patellar tendinopathy (not Patellar) (line 38).

Thanks for the comment, it was fixed.

Rephrase the first two sentences of the second paragraph, as the grammar is not very accurate.

Thanks for the comment, they were rephrased as follows: “As the only interface between the foot and the ground, Footwear plays an important role in gait biomechanics and is of great concern in the management of lower limb injuries [4]. Amongst various types of footwear, boots are often used in rehabilitation, sports, and occupations to support and protect the foot and ankle on rough terrain and environmental conditions [5,6].”

Replace Sinclair and his colleagues with Sinclair et al.

Thanks for the comment, It was fixed as requested.

Name the studies the authors refer to (line 62).

The names of the studies were as follows: “Cikajlo and Matjačić (2007) and Böhm and Hösl (2010)”.

Discussion. For the referring studies, add the year of publication in brackets (for example, Sinclair (2014)).

Thanks for the comment, this one and the other instances were fixed.

Explain what boots literature means.

It was modified to “boot-related studies” which I think is a better term and is similar to footwear-related studies.

Name the prospective studies the authors refer to (line 269).

The name of the study was added to the text as follows: “Theisen et al. (2016)”.

Specify the difference between the number of the participants of the current study and the similar ones.

We analyzed the running biomechanics of 17 participants but the number of participants in similar studies by Sinclair et al. (2014, 2014, and 2015) was 13, 12, and 10 participants, respectively. The following words were added at the end of the counterpart sentence: “, which were less than 14 subjects”

Point out the clinical implications of the current study.

Within the text, we frequently mentioned the relationship between our findings and musculoskeletal disorders (ACL injuries, patellofemoral pain, and tibial stress fracture), as well as degenerative joint diseases (hip and knee osteoarthritis). Also, the following sentence was added: “Additionally, the alterations that occurred in lower limb joint biomechanics during running with boots may be clinically significant for people who frequently use boots for occupation, sport, or rehabilitation. Long-term usage of boots may increase the risk of ACL injury as well as hip and knee OA”.
